# The Government and the Voluntary Hospitals

**Published:** 1912-11-23

**Authors:** 


### The Hospital

The Workers' Newspaper of Administrative Medicine and Institutional Life, Administration, National Insurance and Health.

No. 1377, Vol. LIII. SATURDAY, NOVEMBER 23, 1912. PRICE ONE PENNY.

### THE GOVERNMENT AND THE VOLUNTARY HOSPITALS.

Voluntary hospital managers and the Government, unless immediate action is taken, must dangerously drift towards a disaster so terrible in the cruelties it may inflict upon all classes of workers throughout the United Kingdom that the monstrous injustice of it, when the blow falls, may justify, if it does not even cause, a revolution. We showed on a recent occasion that on a moderate computation on and from January 16, 1913, each week some 600,000 sick people who have become insured persons will probably require adequate medical treatment out of the 15,000,000 who under the National Insurance Act have had compulsorily to pay with their employers the necessary premiums, and are so entitled to demand it as a right from the Government. Of this army of 600,000 sick people, it is estimated that at least 30,000 a week will need, for their adequate medical treatment, hospital provision. Before the Act comes into force they have been able to obtain it on a philanthropic basis at the voluntary hospitals, but from January 16, 1913, they can no longer, without degrading them, be treated in any sense as objects of charity. They will, in fact, be free men and women, who are entitled to expect such treatment as a right in virtue of the actual payments made by themselves and their employers. To these, too, it is conceivable that a member of the Government who put politics before statesmanship might claim that the total of 7d. represented by these payments is not adequate to defray the cost of hospital treatment, and that Mr. Lloyd George has never said that it was, or ever spoken of hospital treatment as such in any of his speeches. Assuming for the moment that this view, however callous, may be justified as a matter of figures, we may point out that there has been, in addition, a payment of 2d. a week from the Government for each insured person, that this 2d. will represent a revenue of about £100,000 a week, and that this sum, if available to-day for the direct advantage of every insured person throughout the United Kingdom, would be ample to defray the cost of the hospital treatment of every insured person and to meet the just requirements of the medical profession, which the Government have so far failed to satisfy. In Germany the working classes, through their approved societies, are able to pay for hospital treatment out of the contributions made by their employers and themselves, and for every other requirement which illness may entail upon them and their families. In Germany the medical treatment was not adequate at first to the requirements of the insured, but the German workmen absolutely refused to be degraded by having charity in the shape of free hospital benefit conferred upon them, and the *Kassen*, or approved societies, were speedily put in a position to pay for all the hospital treatment their members might require.

We showed in a previous article (pp. 143-4, The Hospital, November 9) that under Mr. Lloyd George's Act the 2d. a week contributed by the Government has been appropriated (*i.e.*, diverted) for many years to create a fund to make his scheme financially sound. At the expiration of this period, when the Government's contributions become available for equal distribution amongst all the insured persons, it will represent a sum infinitely greater than that required to provide every insured person with hospital and other extra forms of medical treatment and care which they may need. The question arises, Will the British people consent to what their German confrères denominated the degradation of charitable aid forced on them by the Government whenever they are so grievously ill as to require hospital treatment? We do not for one moment believe that they will suffer this degrading treatment, remembering that they and their employers are compulsorily made to pay for National Insurance. What is more, the people's just indignation may be fanned to fever heat when they further realise that, apart from hospital treatment, the actual provision which they can obtain from their approved societies in the day of sickness will not even defray the cost in all circumstances of adequate treatment in their own homes.

Let us next consider the position of the voluntary hospitals *vis-à-vis* with the Government. The Government is credited with this attitude towards these institutions, the working classes, and every insured person who may need hospital care. The Government maintain that to say, on the one hand, that the voluntary hospitals cannot continue to admit as free cases insured persons as in-patients from January 16 next, as they have been taking them for years past, is mere "bluff," and if a reason is wanted for holding this opinion, the silence and inaction of the managers of these great institutions are given. If hospital managers believed that financially the future of voluntary hospitals was imperilled by their accepting as free in-patients all the insured persons who apply to them for admission, the Government contend they would long ago have presented a memorial by a great deputation to drive home the facts and throw the whole responsibility for the disaster, with which the working classes are threatened in this matter, upon the proper shoulders—namely, those of the Government. Will this not be held to be a reasonable view for the Government to take, unless and until the voluntary hospitals unite and, as men of business and men of feeling, speak out in earnest protest against the callous indifference with which the Government are treating this vital question of supreme urgency to the very lives of the workers, and to the preservation of their homes and families, in the hour of grave disablement from sickness? We urge these facts upon the attention of every individual hospital manager and the committee of each voluntary hospital throughout the United Kingdom, because they rest upon knowledge, and that knowledge proves that we have not advanced a single statement which is not founded upon fact. The authorities of certain hospitals have, we know, held their hand pending the decision of the medical profession upon the new position created by Mr. Lloyd George in the latest proposals submitted for their consideration. That decision has now been arrived at, and every hospital committee must take its courage in both hands and act with resolution and directness, or share the whole responsibility with the Government for the miseries which may result from the causes which we have already fully set forth. Apart from the patients and their claims, the hospitals must be fully alive to the financial dangers with which they are threatened if they go on drifting without resolute action until January 16 arrives.

But, apart from the Government's responsibility to the voluntary hospitals and the wanton waste of money which may result from their failure to enter at once into a business arrangement with these institutions on a *pro rata* basis, with provision for the erection of adequate additional accommodation as it may be required, as we fully explained in The Hospital of the 9th instant, the present Ministry in default of such arrangements will be *vis-à-vis* with an army of dangerously ill workers up and down the country, numbering in all probability something like 30,000 fresh cases every week, who may need and most of whom may not procure hospital accommodation. This is the great fact which it is the duty of the voluntary hospitals to urge upon Mr. Lloyd George and the present Government. They should press it with all their might, and without delay, for otherwise they will incur a responsibility which is immeasurably increased by the knowledge which they actually possess, but which Mr. Lloyd George will not admit, that such an absence of hospital provision must mean disaster and death to thousands of people who otherwise, in God's good providence, under existing circumstances would have been restored to health, and continued as the mainstay and backbone of the wives and children dependent upon them. The awfulness of the present position, to those who have knowledge and imagination, makes every thinking man amongst us wonder what can be wrong with Mr. Lloyd George, who seems by his immersion in politics to have shed not only good sense but the very attributes of manhood, by this callous disregard to the dreadful and continuous risks involved by his reckless forcing through of this National Insurance Act before it was thought out in all its bearings, and before due provision had been made to meet the absolute necessities, when ill, of the 15,000,000 insured persons created under it. Have any body of men in the whole history of our nation ever been provided with more convincing facts than those known to the managers of the voluntary hospitals, which, in simple language, could be urged with such telling force as to compel any Government, anywhere, to pause and halt before they allow January 16, 1913, to arrive without one hospital bed being provided in this country on a business basis for the reception of insured persons? Here, again, hospital managers must recognise that to remain inactive any longer is, so far as they are concerned, to join hands with Mr. Lloyd George and the Government in shouldering the responsibility of the disasters which, as sure as night follows day, lie in front of the working classes of this country under the National Insurance Act as matters stand to-day.

